# Fish in the matrix: motor learning in a virtual world

**DOI:** 10.3389/fncir.2012.00125

**Published:** 2013-01-25

**Authors:** Florian Engert

**Affiliations:** Harvard UniversityCambridge, MA, USA

**Keywords:** fictive locomotion, virtual environments, zebrafish, bioluminescence imaging, closed-loop system

## Abstract

One of the large remaining challenges in the field of zebrafish neuroscience is the establishment of techniques and preparations that permit the recording and perturbation of neural activity in animals that can interact meaningfully with the environment. Since it is very difficult to do this in freely behaving zebrafish, I describe here two alternative approaches that meet this goal via tethered preparations. The first uses head-fixation in agarose in combination with online imaging and analysis of tail motion. In the second method, paralyzed fish are suspended with suction pipettes in mid-water and nerve root recordings serve as indicators for intended locomotion. In both cases, fish can be immersed into a virtual environment and allowed to interact with this virtual world via real or fictive tail motions. The specific examples given in this review focus primarily on the role of visual feedback~– but the general principles certainly extend to other modalities, including proprioception, hearing, balance, and somatosensation.

There are two fundamentally different forms of sensory information that are being processed by the brain. The form that is more commonly studied – also the form that neuroscientists mostly worry about – is the kind that informs the brain about what is happening in the outside world. This kind of information is represented by neural activity that is evoked by changes in the environment due to all possible kinds of physical or biological events. We live, after all, in a constantly changing world and it clearly helps to be informed speedily of these changes. A large part of neuroscience is involved with the study of how this kind of sensory evoked activity is represented at different stages of processing in the brain and how it gets filtered for optimal extraction of the information that is most relevant for the generation of adaptive behaviors.

The zebrafish is a good model system to address these kinds of questions, since its translucence and small size makes it ideally suited for monitoring neural activity throughout the brain with modern optical methods. This striking advantage features prominently in other articles in this special issue and there are multiple examples across many modalities where such studies have added to our understanding of how sensory information is represented in the brain (Niell and Smith, 2005; Ramdya and Engert, 2008; Sumbre et al., 2008; Del Bene et al., 2010; Blumhagen et al., 2011; Grama and Engert, 2012) and how this neural activity ultimately leads to the generation of specific behaviors. Thus, fish have been shown to turn in specific directions with specific turn amplitudes (Orger et al., 2008), to modulate their swim speed according to sensory input (McLean et al., 2008), and to change the threshold for escape turns according to situational context (Mu et al., 2012).

The topic of this review is not related to this kind of question at all. Rather, it addresses the issue of how the second form of sensory information gets processed, namely the kind of sensory activity that results from the motion of the animal itself. Such self-generated sensory stimuli are termed reafference and they occur across many modalities whenever any movement is executed. When walking forward we experience reverse optic flow, that is, we perceive the world to be moving in the opposite direction. We also experience pressure on the bottom of our feet and air might flow over our skin. Whenever we vocalize we experience a very distinct auditory reafference, namely the sound of our own voice which, of course, needs to get processed quite differently than somebody else’s utterance and such reafference clearly is a useful thing to pay attention to when we learn to sing or speak. I’m sure we can, with some creative thinking, even come up with good examples of olfactory reafference.

The main difference between this reafferent signal and the initially mentioned form of sensory input, commonly known as the exafference, is that it does not inform us about what effect the world has on us, but rather tells us what effect we have on the world. As such it informs the brain about the success and accuracy of ongoing movements and is immensely useful for – and most likely central to – all forms of motor learning and motor adaptation. The easiest way one can imagine such a learning process to take place, is that the reafference gets compared, somewhere in the brain, to an expected value, most likely represented by an efference copy, that is, a copy of the motor-command that is usually available in many brain regions. As soon as a difference is detected between expected outcome and actual reafference, plasticity mechanisms need to kick in, in order to adjust future motor-commands.

It is clear that such motor learning phenomena cannot be studied in paralyzed – and much less in anesthetized – animals, since here the actual execution of a behavior is the origin and cause of sensory stimulation.

If the goal is then to study the neural dynamics underlying these reafferent signals, a way has to be found that allows the monitoring of neural activity, ideally at cellular resolution and throughout the whole brain, while the animal is interacting with its environment. An additional requirement for such an experimental set-up is that it ought to allow control over the reafferent signal. In order to do this, an explicit decoupling of the motor action from the resulting sensory feedback is required such that the feedback link can now be programed in under complete experimental control. In such a setting, the subject can be rendered stronger or weaker than in real life by modifying the gain of the motor to sensory transformation. This is a feature that usually comes for free in all virtual environments where the simulated speed and strength of the operator/subject can be dialed in at will.

Such virtual environments usually require the tethering of the animal – and elegant implementations of this approach, where the tethering of the animal has been made compatible with 2-photon imaging, have been described for fruitflies suspended in midair in flight-simulators (Maimon et al., 2010; Tang et al., 2004) or walking on two dimensional treadmills (Seelig and Jayaraman, 2011) and in rodents running in place on floating styrofoam balls (Dombeck et al., 2007; Harvey et al., 2009) (**Figures 1A**,**B**). A major challenge in zebrafish research has long been to design a similar paradigm around a small animal wriggling in water.

**FIGURE 1:**
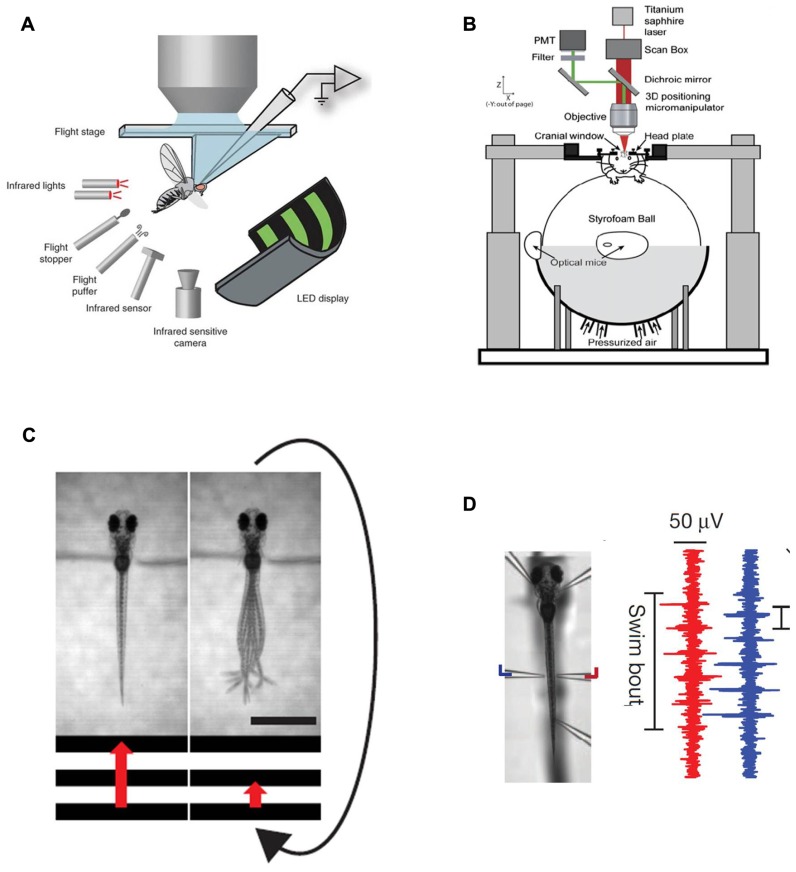
**Existing methods for simultaneous behavior and neural recording.** **(A)** a fly in a flight arena while whole-cell recordings are made from VS cells (from the Dickinson lab, Maimon et al., 2010, reproduced with permission). **(B)** A mouse walking on a suspended ball through a virtual reality environment while its brain is two-photon scanned (from the Tank lab, Dombeck et al., 2007, reproduced with permission; Harvey et al., 2009). **(C)** Diagram illustrating the closed-loop experimental setup in a larval zebrafish. A moving grating is shown to a head-restrained larva (the grating speed is represented by the red arrow) and its behavior is monitored with a high-speed camera. When the fish swims the stimulus slows down such that the relative motion between the larva and the moving grating resembles freely swimming conditions. The scale bar at the bottom right is 1 mm. **(D)** *Left*: Photomicrograph of a fish suspended in mid-water from five pipettes, two of which double as recording electrodes. *Right*: Example of a two-channel recording of a fictive swim. The left (blue) and right (red) signals are out of phase, as in earlier fictive swimming publications such as Masino and Fetcho (2005).

The first step to implement such a paradigm is to establish technology that allows the readout of behavior in immobilized or at least head-fixed preparations, and this has been solved in various ways in the past. One, reasonably straightforward, route is first to embed the fish in low-melting-point agarose (low-melting-point such as not to boil the animal when immersing it into the still liquid medium), then to free the tail once the agarose has set and subsequently observe tail motion with a high-speed camera to obtain a proxy of intended locomotion (**Figure 1C**). This approach has been used in a number of imaging – as well as perturbation studies in the zebrafish larva (O’Malley et al., 1996,^2004^; Ritter et al., 2001; Szobota et al., 2007; Sumbre et al., 2008; Wyart et al., 2009). An alternative approach is to paralyze the animal with a toxin that specifically blocks the neuromuscular junction (substances like curare or bungarotoxin are commonly used), then suspend it in mid-water with several suction pipette and have two or more of the pipettes double as recording electrodes to measure nerve root activity through the skin on both sides of the body (**Figure 1D**). These nerve recordings have been used extensively in lamprey as a readout for fictive swimming (Fagerstedt et al., 2001) and have also lead to exciting findings on midbrain circuitry in goldfish (Fetcho and Svoboda, 1993) and zebrafish (Masino and Fetcho, 2005). Such recordings provide very similar information to tail motion monitored with a camera and provide the additional benefit of removing all possible motion artifacts. One residual, but significant concern associated with these paralytica is of course that they might also interfere with processing at the level of the CNS. As such it is recommended to bolster all experiments that involve fictive swim recordings with thorough controls that ensure that central processing is not compromised by the neurotoxins. One possible way to do this is to perform comparable experiments in non-paralyzed preparations – head-fixed, but tail-free for example – and deal with the resulting motion artifacts through enhanced image analysis (Dombeck et al., 2007).

To make the leap from providing a simple read-out of behavior, be it fictive or physical, to a set-up where the animal actually interacts meaningfully with the environment, another essential step is necessary: the behavior needs to be analyzed in real time and fed back into a computer system that updates the environment (usually a virtual one) according to the recorded locomotor events. This classic approach using virtual environments is well known to all users of flight simulators and all players of first person video games such as Quake or Doom. In the absence of a closed-feedback loop, the delivery of sensory information to the animal is decoupled from the behavior and analysis of true interaction or navigation is not possible. The active player of a video game would then become a passive watcher of television.

Probably the first implementations of such closed-loop systems are described by Bernhard Hassenstein and Werner Reichardt in the 1950s (Hassenstein and Reichardt, 1956), where a rüsselkäfer (*Chlorophanus viridis*) walks interactively on a spangenglobus (**Figure 2**), an ultra lightweight globe made of bamboo twigs (Hassenstein, 1991). This closed-loop preparation was famously used to generate the still valid Hassenstein–Reichardt model for the fundamental computations underlying direction selective responses in the visual system.

**FIGURE 2:**
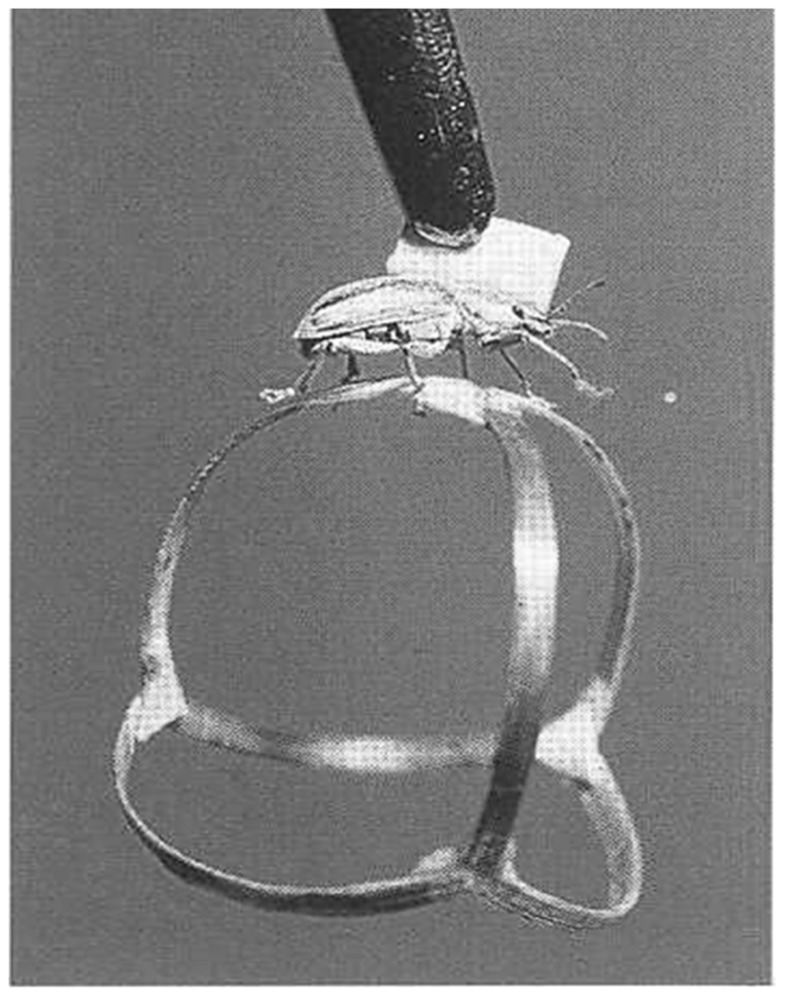
**Tethered *Chlorophanus* walking on a Y-maze globe.**

In order to apply such a closed-loop system to the larval zebrafish, locomotor events need to be analyzed online and the computed locomotion must then be used to update a virtual environment displayed on computer screens placed either below or around the animal. Importantly, the gain in this virtual navigation setting can be dialed in by the experimenter. A given locomotor readout can be translated into a large or small distance covered in the virtual world and thus the animal can be equipped with virtual superpower or virtual feebleness at the dial of a button. Both ways of implementing swimming in a virtual world, fictive swims as well as actual tail motion in a head-fixed preparation, have been used recently in two articles that described the ability of larval zebrafish to adapt to these gain changes. In both studies larval zebrafish were immersed into virtual environments and the fictive strength of the fish – represented by the feedback gain of the closed-loop system – was changed periodically between high and low settings. In both cases it was found that fish indeed change their swimming behavior in response to such changes in the biophysics of the virtual environment (Portugues and Engert, 2011; Ahrens et al., 2012).

One of the main contributions that these studies provided was the development of efficient algorithms that allow the translation of locomotor activity into intended movement of the animal and subsequently the real time update of a virtual environment that was represented by computer monitors surrounding the fish. Particularly in the case where motor nerve recordings serve as the exclusive source of behavioral output, analogies to the movie “The Matrix” are obvious: the feature film describes a world in the distant future where the heroes interact with an entirely virtual world simply by virtue of activity in their brains.

It should be noted that in most of the preparations described in this mini-review, closed-loop feedback has been restricted to vision. In principle it is possible to add other modalities and immerse the animal into a more complete virtual world. The inner ear or the neuromasts of the lateral line could be stimulated whenever a swim event occurs to mimic locomotor-feedback in the form of acceleration or water flow; the tail could be moved passively to provide proprioceptive feedback and one could also change the cues related to temperature and/or chemo-sensation in correlation with the position of the animal in the virtual world. An interesting observation is that in most cases such a complete representation of reafference is not necessary for appropriate and meaningful behavior in a virtual world. Often it is sufficient to provide meaningful and consistent feedback to a single modality and then the absence of feedback in remaining input channels gets quickly ignored.

Good examples for such phenomena are found in current attempts to develop brain machine interfaces that allow monkeys as well as human subjects to move cursors over computer screens, or operate machinery simply by thinking about it. These serve probably as the best examples for the necessary plasticity in such closed systems since here the brain has very little a priory information of how activity in specific neuronal ensembles leads to changes in the environment via the motor systems that connect the two.

As such it is obvious that the brain needs to learn how to control the environment through these novel means, presumably via established algorithms of motor learning.

An intriguing finding in the zebrafish studies – as well as the preceding experiments on flies – was that animals are able to adapt their behavior to different conditions of the virtual environment with surprising speed. Very similar adjustments to artificially induced changes in reafference were found in a series of landmark studies in the weakly electric fish. Here changes in the reafference of the animal’s electric discharge was found to be canceled precisely by a negative image presynaptic to secondary sensory neurons – and this cancellation adapted quickly when the strength of the reafference was artificially manipulated (Bell, 1981; Bell et al., 1997). Furthermore, this adjustable subtraction of an expected value from the actual reafference was not limited to weakly electric fish; similar adaptations were found in many ray finned fishes that are equipped with sensitive electro receptive organs where reafferent signals are generated by various forms of rhythmic muscle activity like breathing or swimming (Bodznick et al., 1999; Zhang and Bodznick, 2008).

Analogously, in a swimming zebrafish a change in the “strength” of the virtual fish, e.g., a scenario where the fish suddenly found itself with much more – or much less – power than expected, the animals responded within a few seconds by adapting their behavior: a “weak” fish, for instance, increased its swim vigor to compensate for the decrease in strength of the visual feedback, whereas a “strong” fish did the opposite. Interestingly, the animals also “remembered” these changes in behavior for some time.

Since fish are readily amenable to whole brain calcium imaging – as is made clear in several other articles in this issue – it was straightforward to isolate several different types of neural activity that occurred during this behavior (Ahrens et al., 2012). Some neurons increased their activity when the fish swam harder, others when the fish swam more gently. Yet other groups, arguably the most interesting ones, were specifically active during the period when reafferent feedback was changed to render the animal unexpectedly weak or powerful. These “error” or “surprise” neurons are good candidates for the sites in the fish’s brain where an efference copy gets compared to the reafference and they were found in many different brain areas, including the inferior olive and the cerebellum, both areas known to be involved in motor control in mammals. While it is still unclear how far the similarities between larval zebrafish’s and mammalian brains extend in terms of anatomy and neuronal cell-types, it is clear that the general principles are pretty much conserved. Essential elements, like the inferior olive, the different nuclei of the cerebellum as well as individual neuronal types such as Purkinje and granule cells are certainly present in both species.

As such these studies open the field for a whole array of experiments that hopefully will shed light on the neural basis of motor learning in the vertebrate brain.

To summarize, these closed-loop implementations of fish behavior in virtual environments allow first forays into the study of entire neural ensembles, spanning from sensory input all the way to motor output, in a behaving animal that is flexibly adjusting its behavior in responses to changes in the feedback it receives from the environment. It thus opens the way for many similar experiments in which we can exhaustively study neural activity during true interactive behavior in a vertebrate model organism. Hopefully, this will serve to illuminate how large populations of neurons, across many brain areas, work together to generate flexible behavior.

## Conflict of Interest Statement

The author declares that the research was conducted in the absence of any commercial or financial relationships that could be construed as a potential conflict of interest.
